# Subtropical Potential Vorticity Intrusion Drives Increasing Tropospheric Ozone over the Tropical Central Pacific

**DOI:** 10.1038/srep21370

**Published:** 2016-02-12

**Authors:** Debashis Nath, Wen Chen, Hans-F. Graf, Xiaoqing Lan, Hainan Gong, Reshmita Nath, Kaiming Hu, Lin Wang

**Affiliations:** 1 Center for Monsoon System Research, Institute of Atmospheric Physics, Chinese Academy of Sciences, Beijing 100190, China; 2 Center for Atmospheric Science, University of Cambridge, Cambridge, UK

## Abstract

Drawn from multiple reanalysis datasets, an increasing trend and westward shift in the number of Potential Vorticity intrusion events over the Pacific are evident. The increased frequency can be linked to a long-term trend in upper tropospheric equatorial westerly wind and subtropical jets during boreal winter to spring. These may be resulting from anomalous warming and cooling over the western Pacific warm pool and the tropical eastern Pacific, respectively. The intrusions brought dry and ozone rich air of stratospheric origin deep into the tropics. In the tropical upper troposphere, interannual ozone variability is mainly related to convection associated with El Niño/Southern Oscillation. Zonal mean stratospheric overturning circulation organizes the transport of ozone rich air poleward and downward to the high and midlatitudes leading there to higher ozone concentration. In addition to these well described mechanisms, we observe a long-term increasing trend in ozone flux over the northern hemispheric outer tropical (10–25°N) central Pacific that results from equatorward transport and downward mixing from the midlatitude upper troposphere and lower stratosphere during PV intrusions. This increase in tropospheric ozone flux over the Pacific Ocean may affect the radiative processes and changes the budget of atmospheric hydroxyl radicals.

Climatologically, subtropical Potential Vorticity (PV) intrusions or Rossby wave breaking events occur predominantly within the equatorial westerly ducts, which show large interannual variability over the Pacific and Atlantic sectors^1^,^2^,^3^,^4^. The westerly ducts can be defined as the regions of upper tropospheric equatorial westerly wind over the Pacific and Atlantic Ocean. The frequency of these intrusion events is higher during boreal winter (December-March, >75%), when there are stronger Rossby waves in the midlatitudes that potentially can extend to very low latitudes, and over the Pacific Ocean (150°–280°E), compared to any other season and anywhere else. These wave breakings can be seen in maps of PV. From the climatological analysis with 20 years of NCEP/NCAR reanalysis data it was shown^4^ that the interannual variability in Pacific intrusion events correlates highly with the phase of El-Niño/Southern Oscillation (ENSO). There are fewer intrusions in the warm (El-Niño) and more during the cold (La-Niña) phase with weaker and stronger westerlies, respectively. Owing to the impact of El Niño/Southern Oscillation (ENSO) on Pacific upper troposphere (UT, 200 hPa) flow, several studies have explored the link of ENSO with Rossby wave propagation into the tropics^5^,^6^.

As mentioned above, regions of UT westerly winds are the preferred locations for intrusions of extratropical air deep into the tropics^1^. Therefore, any long-term trend in PV intrusions might be closely linked with changes in background zonal wind conditions i.e. UT equatorial westerlies. Moreover, subsequent variation in the subtropical jet with stronger diffluence in the jet exit region further amplifies the frequency of the events. Weakening of the ambient westerly winds allow the waves to compress in longitudinal scale and grow in latitudinal scale. They then overturn or break eastward of the Asian jet exit region. The stronger the Subtropical jets (STJ), the fewer waves reach the inner tropics and break there. If the STJ is weak, more Rossby waves can penetrate deep into the tropics and then break at the strong equatorial westerly duct.

However, the variability in UT zonal wind tied closely with the changes in SST anomalies, particularly in the Pacific Ocean^7^,^8^. The decadal trend in DJFM mean SST anomaly exhibits a gradual warming pattern stretching from the western Pacific to the warm pool with two subtropical flanks of warming on either side of the equator, constituting a horseshoe pattern^9^ of warming. On the other hand, the cooling trend extending from the equatorial central to the eastern Pacific^5^,^7^,^8^,^10^,^11^, constitutes a strong zonal gradient. It drives an enhanced convective activity in the Western Pacific that leads to precipitation, and the latent heat released in the process strengthens the Pacific Walker circulation. This process strengthens the near equatorial UT westerly duct and is part of the Pacific Walker circulation. The interannual variability of the Pacific Walker circulation is closely related to ENSO^12^,^13^,^14^, while its multidecadal strengthening is in line with global warming^15^. Contrastingly, most of the coupled models advocate a weakening^9^,^16^,^17^ of Walker circulation in the recent decades. However, few argued that the recent multidecadal changes in Pacific SSTs are connected with the negative phase of Pacific Decadal Oscillation (PDO)^18^.

On the other hand, the meridional circulation behaves differently in different regions. In the Pacific, Hadley circulation consist of two cells, one in the western and another in the central-eastern Pacific, with warm and moist air rising in the intertropical convergence zone (ITCZ), then diverging northward and southward in the upper troposphere, and descending over the subtropical highs^19^. In boreal winter the ITCZ is closest to the equator but in northern hemisphere^20^,^21^. Theoretically, the upper branch of the Hadley circulation provides the necessary forcing to drive the STJ. The cooling trend over central to eastern Pacific suppressed the convective activity due to sinking air motion. This process weakens Central-Eastern Hadley circulation and the STJ, due to the weakening of poleward divergent flow in the UT^19^,^20^,^21^. Therefore, the long term variability of the equatorial UT westerly winds and STJ are closely associated with the strength of Walker and Pacific Hadley circulations, induced by spatial variations in the SST warming/cooling patterns over the Pacific Ocean. However, the Walker and regional Hadley circulations alone cannot account for the changes in UT zonal wind. Additional factors like local heating anomalies too have affected the processes and are discussed more in the result section later.

Ozone is of great importance to the climate due to its radiative effects^22^. It is the primary source of the hydroxyl radical (OH) which acts as an oxidizing agent in the atmosphere. Near the surface, ozone is a regional pollutant that is harmful to human health and vegetation^23^. Due to long-term changes in anthropogenic emissions (since the 1970’s), the ozone shield depletes in the extratropical stratosphere^24^. Increase in tropospheric ozone or decrease in stratospheric ozone has a significant negative impact on the society. These intrusions have significant impact on the composition of the atmosphere and radiative balance by changing the concentration of ozone in the troposphere^1^,^25^. However, most of the previous case studies using ozonesonde and airborne lidar measurements suggested that stratospheric air masses with high ozone concentration can reach the upper troposphere in the tropical Pacific^26^,^27^,^28^,^29^, but only few studies found its signature in the lower troposphere (LT)^30^,^31^. The existing theory proposes that, the zonal mean stratospheric overturning circulation organizes the transport of ozone rich air poleward and downward to the high and midlatitudes leading there to higher ozone concentration^32^. In addition to these well described mechanisms, we observe a long-term increasing trend in ozone flux over the northern hemispheric central Pacific that results from potential vorticity (PV) intrusions. Despite the significance of PV intrusions (PVI) on the tropical and subtropical dynamics, there are very few studies addressing the climatology of such intrusions related to stratosphere troposphere exchange (STE) and tropospheric ozone distribution.

In the following section, we try to establish the hypothesis, which links the decadal trend in PV intrusion frequencies over the Pacific Ocean with the variation in UT zonal wind. Using multiple reanalysis datasets we investigate the changes in the strength of UT westerly duct and STJ during boreal winter months with possible implications from anomalous warming and cooling over the Pacific Ocean. Finally, we relate the PV intrusion with long-term trend in ozone fluxes over the northern hemispheric central Pacific, which is in stark contrast to the existing mechanism (poleward and downward transport).

## Results

### (a) Variability of PV intrusion events

**Figure 1:**
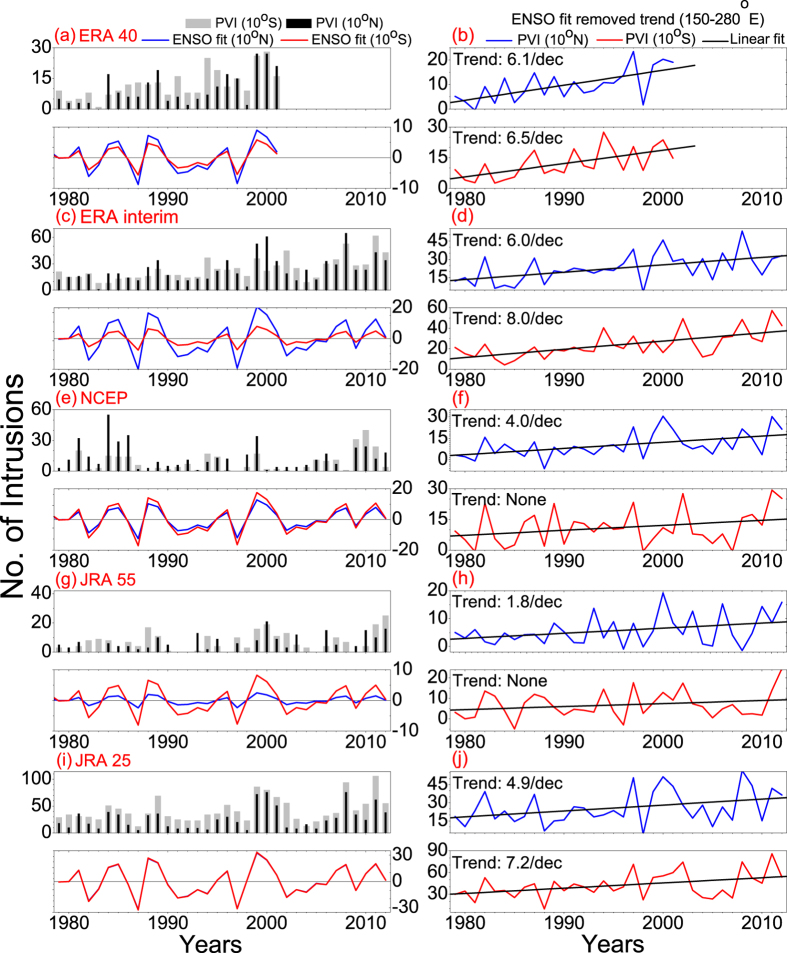
PV intrusion trends at 10°N and 10°S. (**a**,**c**,**e**,**g**,**i**) Represent the number of intrusions/year (PVI) at the 200 hPa/350 K level over the Pacific Ocean (150°–280°E) for ERA40, ERA interim, NCEP, JRA55 and JRA25, respectively. The light gray and black bars correspond to PVI at 10°S and 10°N, respectively (upper panels). The interannual variability in PVI over the Pacific Ocean due to ENSO at 10°N (blue line) and 10°S (red line), are plotted in the lower panels. (**b**,**d**,**f**,**h**,**j**) Represent the ENSO (Nino3.4 index) fit removed trend of Pacific events at 10°N (upper panel, blue line) and 10°S (lower panel, red line) for the datasets mentioned above. The significant (>95%) decadal trends are mentioned in the top left corner of each subplot.

**Figure 2:**
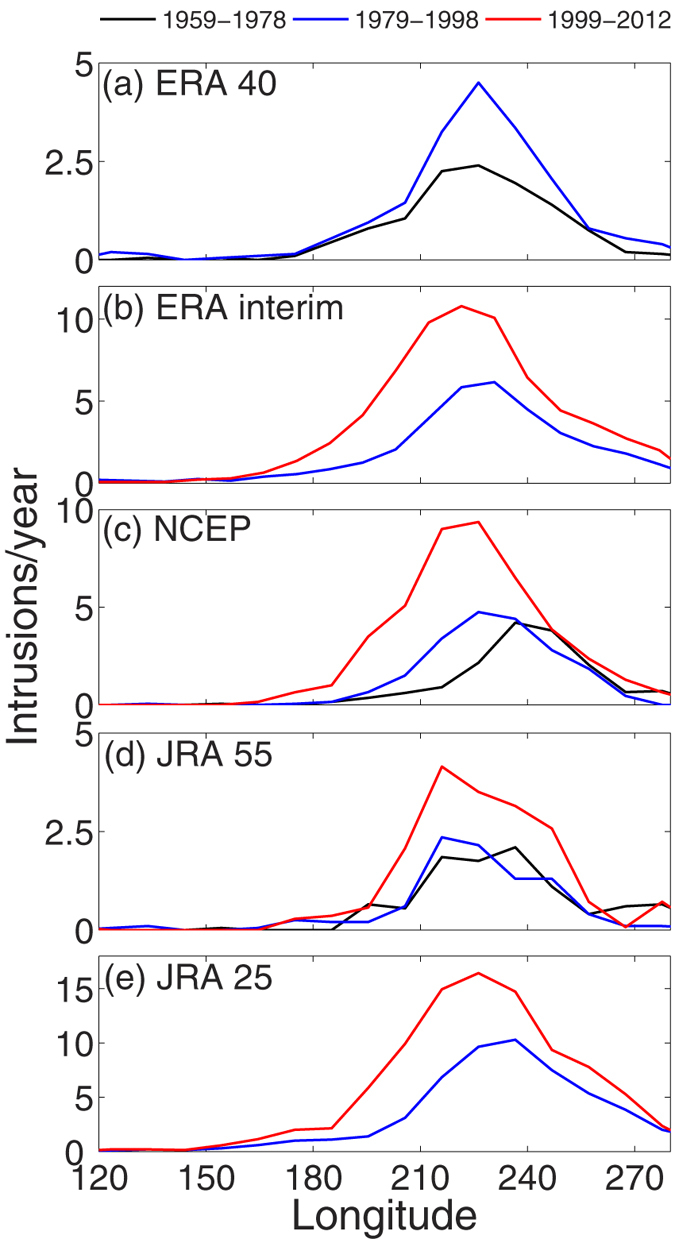
Number of PV intrusions/year for ERA 40, ERA interim, NCEP, JRA 55 and JRA 25 for three periods (1959–1978, 1979–1998 & 1999–2012) are shown in panel (**a**–**e**), respectively.

The number of PV intrusions/year (at 10°N and 10°S) over the Pacific Ocean (Fig. 1) is detected from daily PV fields in ERA40, ERA interim, NCEP, JRA55 and JRA25, based on the criteria discussed in the methods section (PV intrusion). We find that the average number of PV intrusions/year in all five datasets and in three different time periods (1959–1978, 1979–1998 and 1999–2012) exhibits a consistent increasing trend towards the later decades. Moreover, the distribution of PV intrusion events widens zonally and the frequency peak shifts gradually westward toward the central Pacific (Fig. 2).

Significant (>95%) decadal trends in PV intrusion frequency over the Pacific Ocean at 10°N and 10°S are shown in Fig. 1 (upper and lower panels of Fig. 1b,d,f,h,j). Between 1979 and 2012 the results show an apparent increasing trend in all five datasets, at least at 10°N. At 10°N (10°S) the intrusion frequency increases by 6.1 (6.5), 6.0 (8.0), 4.0 (no trend), 1.8 (no trend) and 4.9 (7.2) per decade for ERA interim, ERA 40, NCEP, JRA 55 and JRA 25, respectively. Moreover, despite a monotonous trend (at least at 10°N) a two-fold increase in the intrusion frequency since mid-1990 is noteworthy. Furthermore, the interannual variability strongly correlates with the phase of ENSO (r = –0.67, –0.79, –0.80, –0.71 and –0.85 with Nino3.4 index, for ERA40, ERA interim, NCEP, JRA55 and JRA25, respectively). There are fewer events during El-Niño (1982/83, 1986/87, 1990/91, 1997/98, 2002/03 and 2009/2010), with weaker westerly wind in UT over the equator and stronger STJ. In contrast, there are more PV intrusions during La-Niña years (1981/82, 1984/85, 1988/89, 1995/99, 2007/08 and 2010/2011), with stronger westerly wind in UT and weaker STJ^1^ (lower panels of Fig. 1a,c,e,g,i). Despite a continuous increasing trend the long term tendency in intrusion frequency become clearer once the interannual variability due to ENSO is linearly removed (method section: ENSO fit). The residual time series of the number of events at 10°N and 10°S, which is uncorrelated to ENSO, is shown in Fig. 1. All the trends shown in Figs 1 and 2 and elsewhere in the manuscript exceed at least 95% significance level (two-tailed student t-test). This is the first time report on the decadal trend in PV intrusion frequencies with multiple reanalysis datasets.

### (b) Decadal trend in UT zonal wind

**Figure 3:**
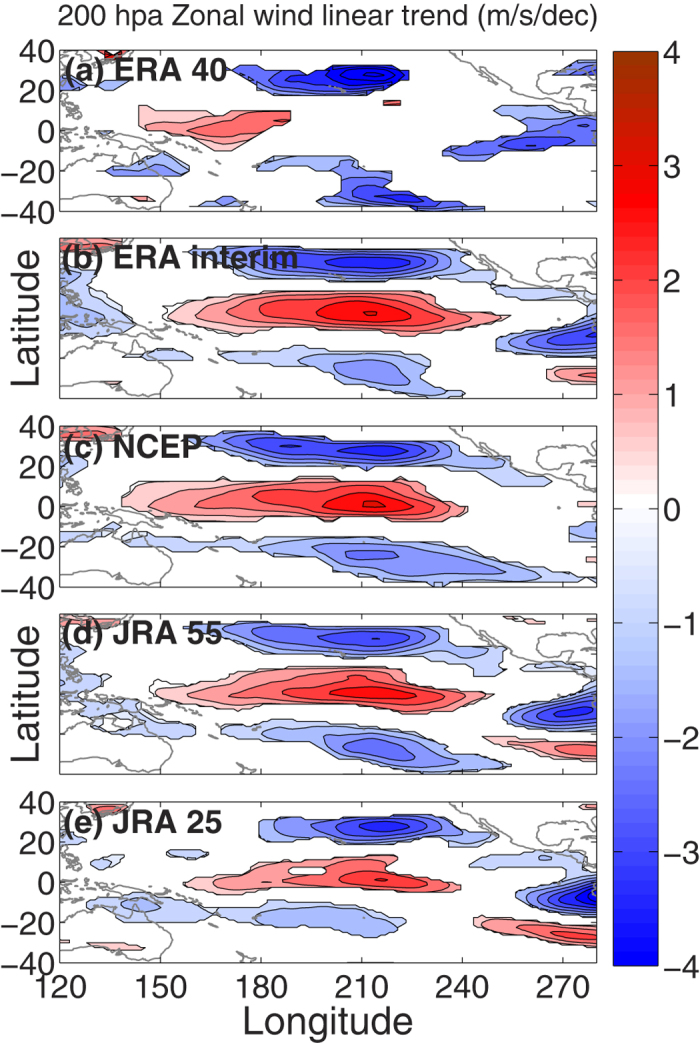
Winter mean (DJFM) linear decadal trend in 200 hPa zonal wind (1979–2002/2012). The panel (**a–e**) represent the linear least square decadal trend in zonal wind (m/s/dec) at 200 hPa for ERA40, ERA interim, NCEP, JRA55 and JRA25, respectively. Values greater than 95% significant level (two tailed student t–test) is shown with color shading. The maps in the figure are generated using the **MATLAB** software (Version: R2012b (8.0.0.783) & URL: http://www.mathworks.com/products/matlab/?s_tid=srchtitle).

To investigate the hypothesis, decadal trends (1979–2002 (ERA 40 only) /2012) in DJFM mean UT zonal wind for ERA40, ERA interim, NCEP, JRA55 and JRA25 reanalysis products are shown in Fig. 3a–e, respectively. A long-term strengthening of the UT equatorial westerly winds and weakening of the STJ over the central Pacific is evident in all five datasets. This trend is also found in other datasets, like the 20^th^ Century reanalysis (1979–2012), MERRA (1979–2012), CFSR (1979–2010) and in an ECHAM5 (1979–2007, Text SM1) climate model simulation forced by HadSST1 anomalies (Figures SM 1a, b, c and d). Overall, the tendency towards stronger UT equatorial westerlies and weaker STJ over the Pacific is very robust across a wide range of reanalysis products in the last quarter of the 20th century. These decadal trends in UT zonal wind are uniquely addressed here and are linked with anomalous changes in SST over the Pacific Ocean.

The near equatorial UT westerly duct is part of the Pacific Walker circulation and its variability is associated with ENSO, PDO and anthropogenic global warming. However, the heating anomalies in the Western Pacific induce a zonal mass circulation with upper tropospheric westerly winds to the east of the Maritime continent (a Walker-like circulation), this circulation alone cannot account for the observed geographical pattern of the climatological westerly duct structure. We will throw more light on it in the discussion part of our manuscript. On the other hand, Figure SM2 exhibits decadal trend in Hadley SST anomalies (color shading) and mean (ERA40, ERA interim, NCEP, JRA 55 and JRA 25) UT divergent wind (arrows). We have found that the regional Hadley cell enhances over the western Pacific (arrows pointing poleward) and weakens over the eastern-central Pacific (arrows pointing equatorward), which is induced by the strong warming over the western Pacific and strong cooling over the central-eastern Pacific, respectively^7^,^8^,^10^,^11^,^28^,^33^ (Figure SM2).

### (c) Decadal trend in Ozone concentration

**Figure 4:**
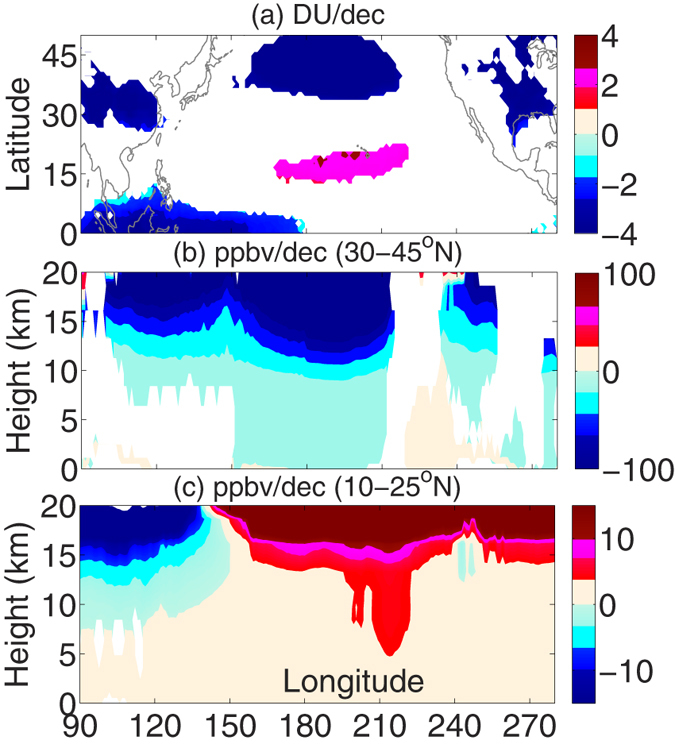
Winter mean (DJFM) linear decadal trend in Ozone. **(a)** Linear least square decadal trend in total columnar Ozone (DU/dec) from TOMS (1983–2012). For the shorter period (2003–2012), the trend is similar and even covers wider region over the Pacific. **(b,c)** Represent the AIRS vertical ozone density trends (ppbv/dec) in the midlatitudes (30–45°N) and outer tropics (10–25°N), respectively. All trends exceed the 95% confidence level based on a two tailed student t–test. The maps in the figure are generated using the **MATLAB** software (Version: R2012b (8.0.0.783) & URL: http://www.mathworks.com/products/matlab/?s_tid=srchtitle).

During boreal winter months we observe an increasing decadal trend (2 to 3 DU/decade) over the outer tropical central Pacific and a decreasing trend (−9 to −10 DU/decade) over the midlatitudes (30–45°N) in total columnar ozone measurements from Total Ozone Mapping Spectrometer (TOMS, 1983–2012, Fig. 4a). The column integrated values are, however, not suitable to estimate vertical transport processes. Therefore, to investigate the vertical trend structure, we utilize the ozone concentration data available from the Atmospheric Infra–Red Sounder (AIRS, 2003–2012). More details about AIRS & TOMS are discussed in Text SM1. Figure 4b,c represent the longitude–height section of linear trends for AIRS ozone concentration from the surface to 20 km altitude as the mean over midlatitudes and outer tropics, respectively. In the midlatitudes the ozone concentration decreases significantly, particularly in the LS, while it increases in the outer tropics. Furthermore, in the outer tropics the trend propagates down to the LT (~5 km) in a narrow band over the central Pacific. The depletion trend in ozone flux over the midlatitudes is much higher than the increase over the outer tropics. In general, stratospheric circulation transports ozone rich air poleward and downward to the high and midlatitudes^32^. Therefore, our results uniquely address the Ozone trend over central Pacific, which is different from the existing hypothesis. Specifically, the increase in total ozone occurs on and around Hawaii^29^, and hence based on the availability of ozonesonde record we performed trend analysis between 1993 and 2012 (Figure SM3). The results exhibit an increasing decadal trend in ozone concentration by 0.5–1.5 ppmv/decade (increasing as a function of height) over Hawaii during the boreal winter months and strongly supplement the analysis from AIRS, at least in the UT and LS region (10–22 km). All the trends exceed at least the 95% significance level (two-tailed student t-test). If we look at Fig. 4c carefully, the downward extension of ozone concentration to LT occurs predominantly around 215^o^E, which is little eastward to Hawaii (205^o^E). This probably explains the absence of any significant trend in the ozonesonde profiles below 10 km over Hawaii.

**Figure 5:**
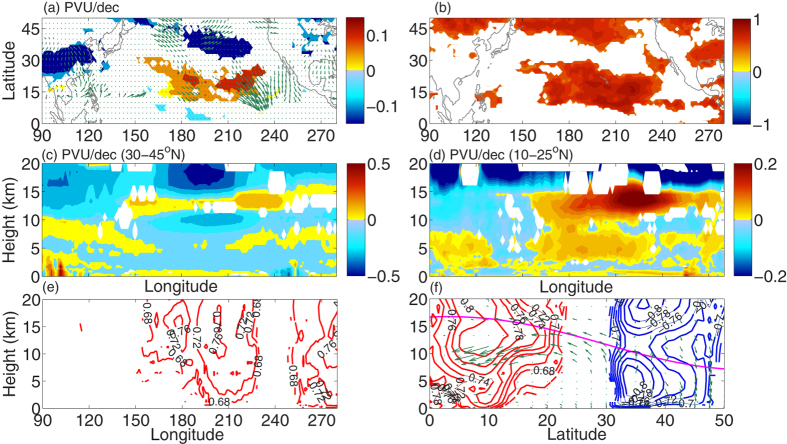
Winter mean (DJFM) linear decadal trend in PV (1979–2002/2012). **(a)** Linear least square decadal trend in PV (PVU/dec) at 200 hPa/350 K level (mean of ERA40, ERA interim, NCEP, JRA55 and JRA25). The green arrows indicate decadal trends (DJFM, 1979–2012) of the horizontal component of Plumb wave activity flux (mean over 70 hPa-200 hPa from ERA interim data), **(b)** correlation coefficient between PV and TOMS ozone. **(c,d)** Represent the vertical PV trends (PVU/dec) for ERA-interim, at (30–45°N) and (10–25°N), respectively. **(e)** Longitude-height (mean over 10–25°N) section of the mean correlation coefficient between Pacific PV intrusion events (ERA interim, NCEP, JRA55 and JRA25) and AIRS ozone density. **(f)** Represents the same as (**e**) but the latitude-height section and mean over 160–240°E. The red and blue curves indicate mean (ERA interim, NCEP, JRA55 and JRA25) positive/negative correlation between PV intrusion events and AIRS ozone density. The green arrows indicate the decadal trend (DJFM, 1979–2012) in a latitude-height section (mean over 160–240°E, ERA–interim data) of Plumb wave activity flux. For the shorter period (2003–2012), the trend in Plumb wave activity flux is comparable. All the trends are significant exceeding the 95% confidence level based on a two tailed student t-test. The maps in the figure are generated using the **MATLAB** software (Version: R2012b (8.0.0.783) & URL: http://www.mathworks.com/products/matlab/?s_tid=srchtitle).

Previous case study shows that intrusion events have only weak signals in total ozone, except January 1987, where the intruded PV anomaly tongue extended deep down to the mid-troposphere^29^. The subtropical intrusions are fairly barotropic and generally do not penetrate into the LT^4^. Figure 5a exhibits DJFM mean decadal trends in UT PV. The increase in PV over the outer tropical central Pacific and the decrease in midlatitudes are consistent with the enhancement and depletion in total columnar ozone (Fig. 4a), respectively. The decadal trend in the horizontal component of the Plumb wave activity fluxes (DJFM and mean over 70 hPa–250 hPa) from ERA interim (1979–2012) data is plotted in Fig. 5a as arrows. The correlation coefficient between TOMS total columnar ozone and UT PV is very high (0.8–1, ≫99%; Fig. 5b). Figure 5c,d exhibit the longitude–height section of linear trends in ERA interim PV, mean over 30–45°N and 10–25°N, respectively. Like for ozone (Fig. 4b,c), the trend is negative over the midlatitudes (UT & LS) and narrow bands of high PV air propagate downward from the UT to the marine boundary layer over the outer tropical central Pacific.

**Figure 6:**
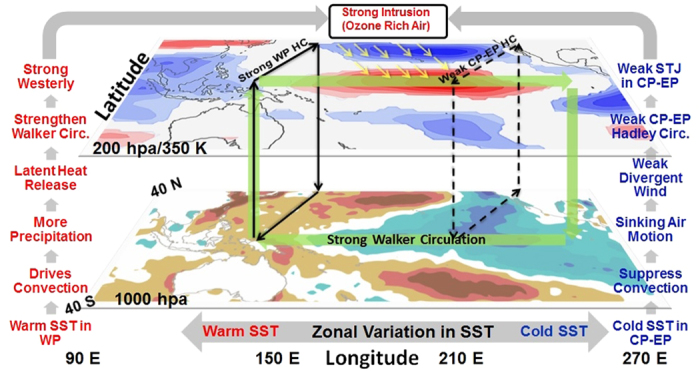
Schematic of the processes involved in increasing ozone flux over the outer tropical central Pacific. It represent the decadal trend in SST anomaly and zonal wind (200 hpa/350 K level), respectively. The strengthening and weakening of western (WPHC) and central–eastern (CP–EP HC) Pacific Hadley cells are shown by bold and dashed loops (black lines), respectively. The strengthened Walker cell over the tropical Pacific is shown as a green loop. Subtropical intrusion of ozone rich high PV air from the mid-latitudes is shown with yellow arrows indicating Rossby wave breakings. The maps in the figure are generated using the **MATLAB** software (Version: R2012b (8.0.0.783) & URL: http://www.mathworks.com/products/matlab/?s_tid=srchtitle).

The linkage between subtropical intrusions and tropospheric ozone is established by performing correlation between PV intrusions and AIRS vertical profiles of tropospheric ozone. It can be seen in longitude–height (mean over 10–25°N) and latitude–height (mean over 160–240°E) sections of correlation patterns. In the former case (Fig. 5e), significant (>95%) mean (ERA interim, NCEP, JRA25, JRA55) correlation is fairly high (0.7–0.75) in the outer tropics. But in the latter case (Fig. 5f), the pattern exhibits strong positive values (0.75 to 0.8, red curve) over tropical central Pacific and negative values (–0.75 to –0.8, blue curve) in the midlatitudes. Comparing the positive and negative correlation patterns in Fig. 5f with the total columnar ozone trend (Fig. 4a), it turns out that the decrease of ozone over midlatitudes (particularly in the UT-LS) is partly (58–64%) due to transport to outer tropics via subtropical intrusion and partly due to long term changes in anthropogenic emissions^24^. The equatorward and isentropic transport due to horizontal intrusions of midlatitude air accounts for ~61–64% of the outer tropical central Pacific ozone variability. The decadal trend in Plumb wave activity fluxes (Fig. 5f, mean over 160–240°E) exhibits an equatorward and downward trajectories from the midlatitudes (UT–LS) to the outer tropics (UT–LT). This facilitates the meridional and isentropic transport of ozone flux across the subtropical tropopause (magenta line, 2 PV unit). A schematic diagram of the processes resulting from the increasing frequency of PV intrusion and its impact on tropospheric ozone over Pacific is shown in Fig. 6.

## Discussions

The results uniquely demonstrate a long-term increase in outer tropical Pacific PV intrusions linked with the strengthening of the upper tropospheric equatorial westerlies and weakening of the STJ. Despite there are some differences among the datasets, all the five datasets reflecting the positive trend in PV intrusion frequency, at least at 10°N. However, the variation in number of intrusions is partially due to the differences in model horizontal resolution and data assimilation schemes and partially due to the differences in diabatic heating. The model resolution differs widely among ERA interim, ERA 40, NCEP, JRA 55 and JRA 25 data, and is T255, T159, T62, T106 and T319, respectively and hence the intrusion frequency. While only ERA interim and JRA 55 adopt the 4DVAR assimilation scheme and others follow the 3DVAR scheme^34^. Moreover, large differences in the distributions of net diabatic heating among different reanalysis datasets, particularly in the UTLS region, too have contributed in the variation of intrusion statistics. Diabatic heating controls heat budget, tracer transport and mixing in the UTLS region. In the UT it implicates transport of tracers from surface to the stratosphere, while the radiative heating in the stratosphere is important for transport and composition in the global stratosphere^35^. Since PV in the UTLS region acts as tracer, its value depends strongly on the magnitude of diabatic heating, particularly in the subtropics.

The tendency towards stronger UT equatorial westerlies and weaker STJ over the Pacific is unequivocal across a wide range of reanalysis products in the last quarter of the 20th century. However, it is worth mentioning in this context that, most of the wind and PV intrusion trend appears to occur in the later decade and this is consistent with the weaker wind trend in ERA 40 (we too have confirmed with other datasets and shorter time period). The increasing width and strength of the westerly duct should allow Rossby wave energy to propagate more deeply southward before breaking because: (a) this may increase the difference between the absolute phase velocities of the Rossby waves and the background wind field, and (b) a wider duct will allow larger zonal wavenumber waves to propagate through the duct. This is in line with the decadal increasing trend in PV intrusion frequencies towards later decades (Figs 1 and 2).

Zonal variation in SST, characterized by gradual warming in the western Pacific–warm pool and cooling in the central–eastern Pacific, is associated with the strengthening of the Pacific Walker circulation. In the Western Pacific enhanced convective activity leads to precipitation, and the latent heat released in the process strengthens the Pacific Walker circulation. It is linked with the trend in global mean temperature, which is related to the emerging anthropogenic greenhouse signal^15^ and negative phase of PDO^18^. However, Walker circulation alone cannot account for the observed geographical pattern of the climatological westerly duct structure. The reason for this is that a very strong component to the maintenance of the global equatorial easterly and westerly wind patterns are due to a quite complicated set of mechanisms including: meridional momentum fluxes^36^, Kelvin-like responses^37^, Rossby responses^38^ and MJO type heating anomalies to upper tropospheric wind patterns^39^.

On the other hand, the central-eastern Pacific cooling trend is linked to the weakening of the central–eastern Pacific Hadley circulation. It suppresses the convective activity due to sinking air motion and imports less angular momentum to the STJ leading to a weakened STJ. However, it is worth mentioning in this context that, the UT divergent wind trend is not collocated exactly with the patterns of SST trends. This is because, the Walker and Hadley circulation alone cannot account for the observed changes in UT wind pattern. The wind structures are the function of additional physical processes beyond the Hadley and Walker circulations that respond to local heating anomalies (e.g. the Rossby and Kelvin responses). Moreover, because the divergence patterns are based on inverting the Laplacian operator, then the wind patterns will be potentially affected by processes that are global in nature (i.e. not simply the local heating anomalies).

While, more PV intrusions result from this weaker STJ on its equatorward side; significantly increase the stratosphere-troposphere exchange processes on the longer timescale. This plays an important role in determining the atmospheric composition, particularly of tropospheric ozone, in the northern outer tropical central Pacific. We observe an increasing decadal trend (2 to 3 DU/decade) over the outer tropical central Pacific and a decreasing trend (−9 to −10 DU/decade) over the midlatitudes (30–45°N) in TOMS data. It may lead to more ozone of stratospheric origin in the LT and even in the marine boundary, which may act as harmful pollutants and affect the radiative processes by changing the global budgets of atmospheric hydroxyl radicals.

There are several questions still remaining unanswered. Will this trend in stratospheric intrusion frequency continue as the climate warms further? Will the trend in UT wind will continue and what are the other factors contribute the changes? How and at what scale will the increase in tropospheric ozone affect the global radiation budget and Pacific climate? These can only be answered by model simulations with long term future projections.

## Methods

### Data used

Trend analysis for PV intrusion is performed on five daily reanalysis datasets at 200 hPa/350 K isentropic levels. The datasets, which assimilate observations with a weather forecast model, include the European Centre for Medium–Range Weather Forecasts (ECMWF) 40 year Reanalysis (ERA40^40^), the ECMWF Interim Reanalysis (ERA interim^41^), National Centers for Environmental Prediction’s Reanalysis (NCEP^42^), Japanese 55 year Reanalysis (JRA55^43^) and 25 year Reanalysis (JRA25^44^). For upper tropospheric wind analysis, along with monthly mean ERA40, ERA interim, NCEP, JRA55 and JRA25, we have used the National Aeronautics and Space Administration’s Modern Era Retrospective Analysis (MERRA^45^), the 20^th^ century Reanalysis (20^th^ Century^46^), NCEP Climate Forecast System Reanalysis (CFSR^47^) and the ECHAM5, the Hamburg version of the ECMWF Global Circulation Model (GCM)^48^. We have used the 350 K isentropic level data, only for the NCEP reanalysis dataset. More details are available from Text SM1.

On the basis of dataset availability the analyses are computed based on 1958–2012 for NCEP, 20^th^ Century and JRA55, 1958–2002 for ERA40, 1958–2007 for ECHAM5, 1979–2012 for ERA interim, JRA25 and MERRA and 1979–2010 for CFSR. For erroneous data quality the first four years of daily PV from JRA55 are not considered in the analysis. Since the daily PV data is available only for ERA40, ERA interim, NCEP, JRA55 and JRA25 reanalysis, we include the corresponding zonal wind data in the main manuscript. However, the additional wind data (MERRA, 20^th^ Century reanalysis, CFSR and ECHAM5) are incorporated for consistency and are shown in supplementary Figure (Figure SM1). The divergent wind is calculated from zonal and meridional wind at 200 hPa for all five datasets (ERA40, ERA interim, NCEP, JRA55, and JRA25) mentioned above. For correlation with Hadley SST and SLP from 1979–2012, all the zonal wind data (9 data products) are interpolated linearly to 360 × 180 and 72 × 37, longitude × latitude grid, respectively.

Total columnar ozone data are available from Total Ozone mapping Spectrometer (TOMS) instruments from 1983–2012 and the data are interpolated linearly to a 360 × 180 longitude × latitude grid. For vertical profiles of volume mixing ratio of Ozone we have used the satellite borne data from Atmospheric Infra–Red Sounder (AIRS)^49^ from 2003–2012 in 360 × 180 × 24 longitude × latitude × pressure grids. More details are available from Text SM1.

### PV Intrusion

Intrusions are defined by first identifying high values of PV (|PV| > 2 PVU, 1PVU = 10^−6^ Ks^2^/kg) at 10°N or 10°S. All such cases within 10° longitude or within 6 days are grouped as a single intrusion event. However, to test the robustness of the climatology, like Waugh and Polvani^1^, the analysis was repeated using 1.75 PVU and 2.25 PVU as the critical value of PV, and the trends were very similar to those presented in this paper except for a slightly greater and lesser number of events, respectively. Small blobs of high PV in the tropics are removed to exclude the cases, which are not immediately related with Rossby wave breaking. The total (along a complete latitude circle) and Pacific intrusions are characterized by the number of events throughout the tropics or in the Pacific Ocean (150°–280°E), respectively.

### Linear fit and trend analysis

The linear trends in PV intrusion, zonal wind, divergent wind, ozone concentration and SST are estimated by a linear fit:


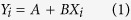


where, *X*_i_ and *Y*_*i*_ is the independent and fitted dependent variable, respectively. *A* and *B* are the intercept and slope, respectively, which can be estimated by the least square method. The decadal trend analysis using reanalysis and model output data are computed based on 1979–2012 for ERA interim, NCEP, JRA 25, JRA 55, MERRA and 20^th^ Century, 1979–2002 for ERA 40, 1979–2010 for CFSR and 1958–2007 for ECHAM5.

### ENSO fit

We calculate a fitted variable, *F(t)*, by:


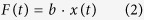


where *b* is the regression coefficient or


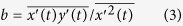


In this manuscript, *y(t)* is the PV trend and *x(t)* is the Niño 3.4 index. A residual time series, which is uncorrelated to *F(t)* and *x(t)*, is computed by subtracting *F(t)* from *y(t)*. The primes indicate the perturbations. However, we note that the residual time series is based on linear and contemporaneous relationships.

### Divergent Wind

The horizontal wind velocity can be divided into non–divergent (rotational) and divergent wind^50^


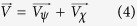


where, 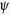 and 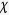 stand for streamfunction and velocity potential, respectively. The horizontal divergence (D) is calculated by


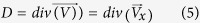


because the rotational components of the horizontal winds make no contribution to atmospheric divergence associated with vertical motion. The velocity potential can be obtained by solving the Poisson’s equation with D. Then the horizontal components of the divergent wind are calculated by,


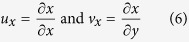


where, 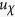 and 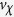 are the zonal and meridional components of divergent wind. In the UT, the zonal component imports the necessary angular momentum to drive the subtropical jet.

### PV tropopause

The dynamical tropopause height is derived from ERA interim reanalysis data with PV = 2PVU unit^31^.

### Plumb Flux

The concept is introduced to analyze the wave propagation from the troposphere to the stratosphere in three-dimensional space^51^. In the log-pressure coordinates the wave activity flux 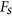 can be represented as follows:


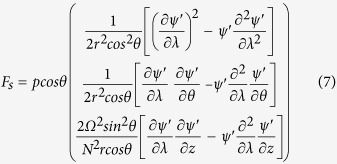


where *ψ*, *λ*, Ω, *z*, and *p* are the streamfunction, longitude, Earth’s rotation rate, altitude, and pressure/1000 hPa, respectively. The primes in the equation 7 represent the perturbation fields.

## Additional Information

**How to cite this article**: Nath, D. *et al.* Subtropical Potential Vorticity Intrusion Drives Increasing Tropospheric Ozone over the Tropical Central Pacific. *Sci. Rep.* **6**, 21370; doi: 10.1038/srep21370 (2016).

## Supplementary Material

Supplementary Information
