# Supplementary material for: Subtropical Potential Vorticity Intrusion Drives Increasing Tropospheric Ozone over the Tropical Central Pacific
Source: Sci Rep. 2016 Feb 12;6:21370. doi: 10.1038/srep21370 (PMC4751467; doi:10.1038/srep21370)
Supplement: Supplementary Information [file srep21370-s1.doc]

**Supplementary Information (SM)**

Subtropical Potential Vorticity Intrusion Drives Increasing Tropospheric Ozone over the Tropical Central Pacific

Debashis Nath^1^, Wen Chen^1*^, Hans-F. Graf^2^, Xiaoqing Lan^1^, Hainan Gong^1^, Reshmita Nath^1^, Kaiming Hu^1^, Lin Wang^1^

**Affiliation:**

^1^ Center for Monsoon System Research, Institute of Atmospheric Physics, Chinese Academy of Sciences, Beijing 100190, China

^2^Center for Atmospheric Science, University of Cambridge, Cambridge, UK

^*^**Corresponding Author:**

Wen Chen

Institute of Atmospheric Physics

Chinese Academy of Sciences

Beijing 100190, China

Ph: +86 010 62551597

e-mail: cw@post.iap.ac.cn

**Keywords:** Intrusion, zonal wind, SST, tropical circulation, Ozone, central Pacific

**Supplementary Figures.**

**
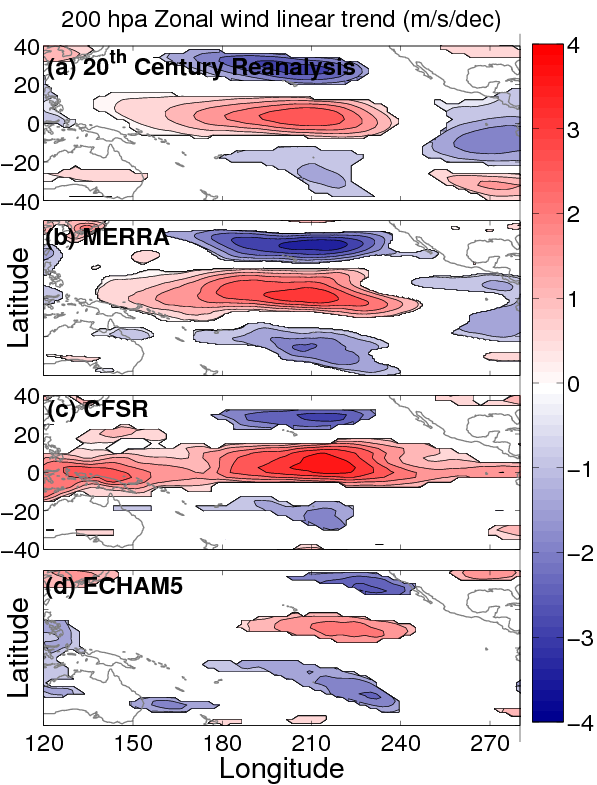
**

**Figure SM1. Winter mean (DJFM) linear decadal trend in 200 hPa zonal wind (1979**–**2012).** The panels (a), (b), (c), and (d) represent the linear least square decadal trend in zonal wind (m/s/dec) at 200 hPa for the 20^th^ Century reanalysis, MERRA, CFSR, and ECHAM5, respectively. Values greater than 95% significant level (two tailed student t–test) is shown with color shading. The maps in the figure are generated using the MATLAB software (Version: R2012b (8.0.0.783) & URL: http://www.mathworks.com/products/matlab/?s_tid=srchtitle).


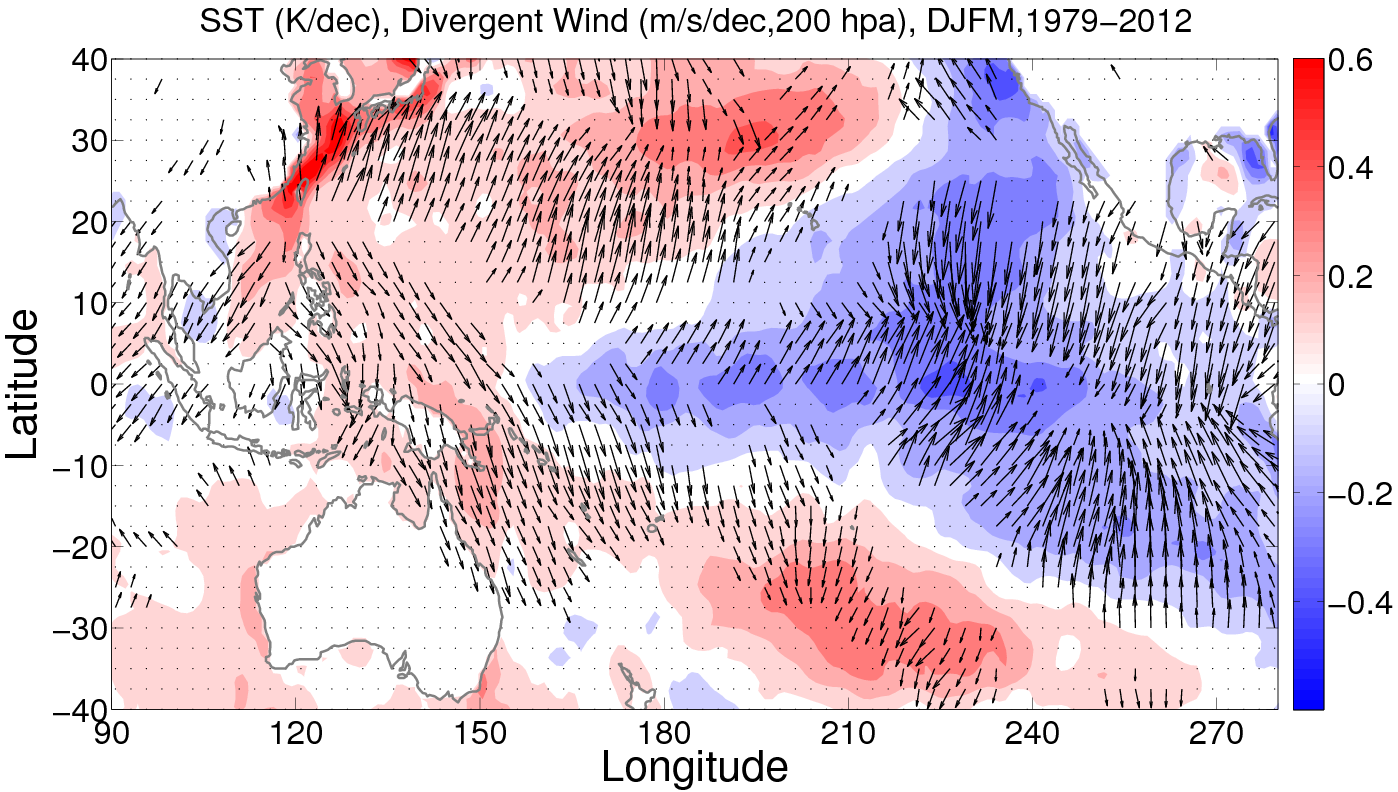


**Figure SM2: Winter mean (DJFM) linear decadal trend in Hadley Centre SST (K/dec) and divergent wind at 200 hPa (m/s/dec).** The red and blue shadings represent positive and negative SST trends, respectively. The arrows represent statistically significant trends in divergent wind at 200 hPa. For the divergent wind the mean trend of ERA40, ERA interim, NCEP, JRA55 and JRA25 is shown. All trends exceed the 95% significant level (two tailed student t–test). The maps in the figure are generated using the MATLAB software (Version: R2012b (8.0.0.783) & URL: http://www.mathworks.com/products/matlab/?s_tid=srchtitle).


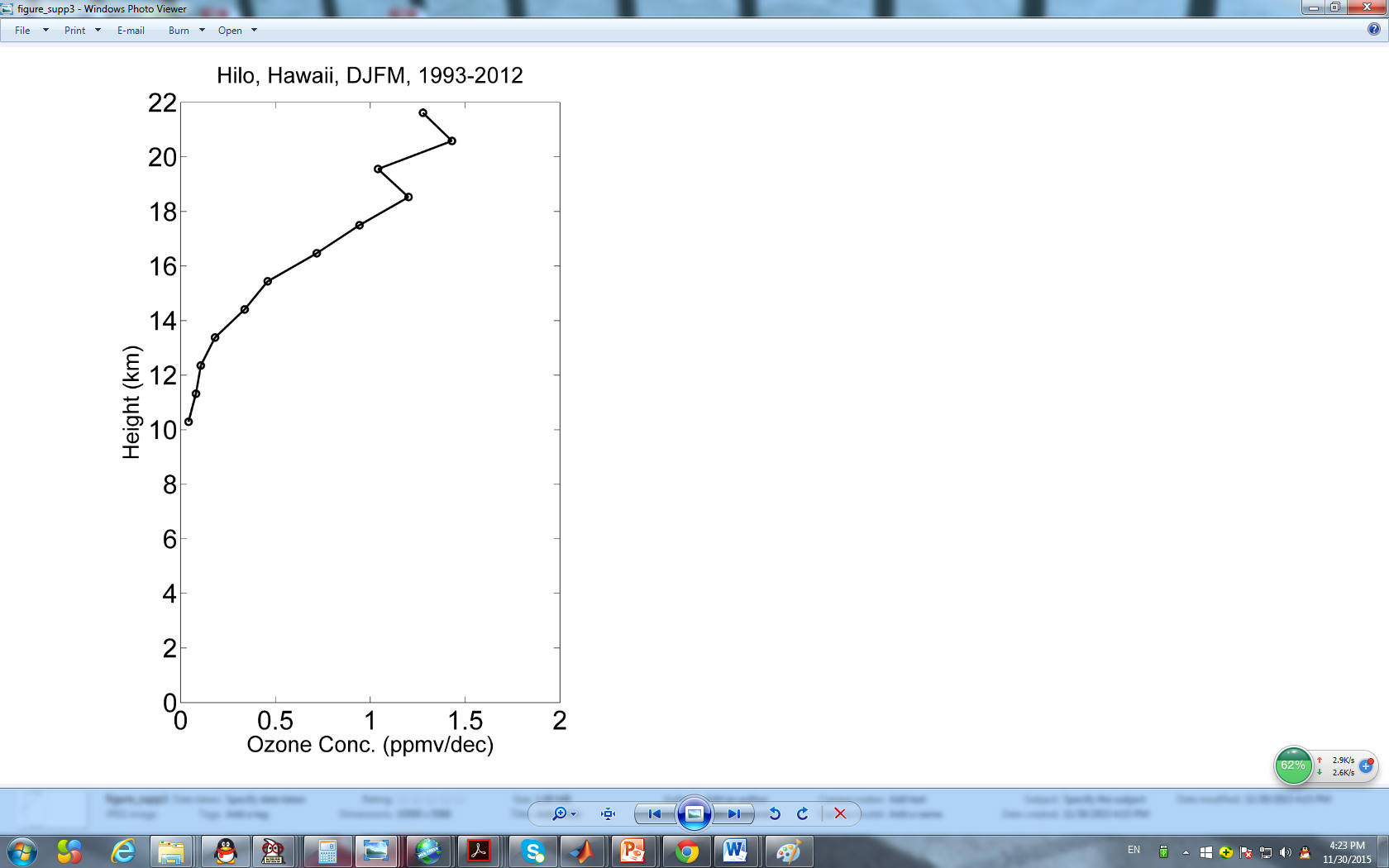


**Figure SM3: Decadal trend in ozone concentration over Hilo, Hawaii.** The decadal trends in DJFM mean Ozone concentration (ppmv/dec) with respect to height over Hilo, Hawaii between 1993 and 2012. The trends exceed the at least 95% significant level (two tailed student t–test).

**Text SM1**

**ECHAM5.** ECHAM5 is the Hamburg version of the ECMWF Global Circulation Model (GCM). ECHAM5 employs a spectral dynamic core. We use a version with triangular truncation at zonal wavenumber 63 (T63) and 19 sigma levels in the vertical^50^. The model is forced by HadISST1, SST and monthly climatological sea ice (SIC) data from the Met Office Hadley Centre. For monthly mean SLP, HadSLP2r is also available from Met Office Hadley Centre. The monthly mean Nino 3.4 index data are obtained from the NOAA Climate Prediction Center.

**TOMS.** Four different Total Ozone mapping Spectrometer (TOMS) instruments were operational at different periods during the 30 years of interest. In our analysis we use total ozone data from *Nimbus–7* (January 1983 to May 1993), *Meteor–3* (May 1993 to November 1994), *Earth Probe* (July 1996 to December 2004), and *OMI* (January 2005–December 2012). There are no TOMS data between November 1994 and July 1996 and the data gaps are filled by linear interpolation.

**AIRS.** In our analysis, we have used the AIRS version 6 monthly means gridded data, which are available from http://disc.sci.gsfc.nasa.gov/. In this version the trend biases in temperature, water vapor, ozone and low altitude clouds have been greatly reduced when compared to ECMWF, radiosondes, and other remote sensing data. The retrieval biases compared to global ozonesondes were less than 5% for both stratosphere and troposphere. The RMS differences are less than 20% in the LS and the troposphere^51^.
